# Gemcitabine Cooperates with Everolimus to Inhibit the Growth of and Sensitize Malignant Meningioma Cells to Apoptosis Induced by Navitoclax, an Inhibitor of Anti-Apoptotic BCL-2 Family Proteins

**DOI:** 10.3390/cancers14071706

**Published:** 2022-03-27

**Authors:** Masahiro Yamamoto, Shuhei Suzuki, Keita Togashi, Asuka Sugai, Masashi Okada, Chifumi Kitanaka

**Affiliations:** 1 Department of Molecular Cancer Science, School of Medicine, Yamagata University, Yamagata 990-9585, Japan; s-suzuki@med.id.yamagata-u.ac.jp (S.S.); ke-togashi@med.id.yamagata-u.ac.jp (K.T.); s-asuka@med.id.yamagata-u.ac.jp (A.S.); m-okada@med.id.yamagata-u.ac.jp (M.O.); 2 Department of Clinical Oncology, School of Medicine, Yamagata University, Yamagata 990-9585, Japan; 3 Department of Ophthalmology and Visual Sciences, School of Medicine, Yamagata University, Yamagata 990-9585, Japan; 4 Research Institute for Promotion of Medical Sciences, Faculty of Medicine, Yamagata University, Yamagata 990-9585, Japan

**Keywords:** malignant meningioma, everolimus, gemcitabine, navitoclax, cellular senescence

## Abstract

**Simple Summary:**

Meningioma is the most common intracranial neoplasm derived from the arachnoid cap cells of the leptomeninges. Malignant meningioma is generally more aggressive than other meningioma and frequently recurs even after surgery and radiation therapy. Clinical trials have been performed on candidate drugs, including everolimus, an inhibitor of mammalian target of rapamycin. However, an effective standard systemic therapy has not yet been established and the prognosis of patients with malignant meningioma is still poor. We recently reported the radiosensitization effects of gemcitabine in malignant meningioma cells, which suggests its potential to enhance the efficacy of candidate drugs for meningioma. In the present study, we demonstrated that gemcitabine enhanced the therapeutic effects of everolimus in malignant meningioma cells, and these effects were further augmented by navitoclax, an inhibitor of anti-apoptotic BCL-2 family proteins, both in vitro and in vivo. The present results provide support for the clinical application of gemcitabine and navitoclax in combination with everolimus to the treatment of patients with malignant meningioma.

**Abstract:**

Despite several clinical trials with encouraging findings, effective standard systemic therapies have yet to be established for malignant meningioma and the prognosis of these patients remains poor. Accumulating preclinical and clinical evidence suggests that gemcitabine is effective against malignant meningioma. To identify drugs with therapeutic effects that may be enhanced in combination with gemcitabine, we screened drugs that have been tested in preclinical and clinical trials for meningioma. In IOMM-Lee and HKBMM malignant meningioma cells, gemcitabine enhanced the growth inhibitory effects of the mTOR inhibitor everolimus, the clinical benefits of which have been demonstrated in patients with meningioma. The synergistic growth inhibitory effects of this combination were accompanied by cellular senescence characterized by an increase in senescence-associated β-galactosidase activity. To enhance the effects of this combination, we screened senolytic drugs that selectively kill senescent cells, and found that navitoclax, an inhibitor of anti-apoptotic BCL-2 family proteins, effectively reduced the number of viable malignant meningioma cells in combination with everolimus and gemcitabine by inducing apoptotic cell death. The suppression of tumor growth in vivo by the combination of everolimus with gemcitabine was significantly stronger than that by either treatment alone. Moreover, navitoclax, in combination with everolimus and gemcitabine, significantly reduced tumor sizes with an increase in the number of cleaved caspase-3-positive apoptotic cells. The present results suggest that the addition of gemcitabine with or without navitoclax to everolimus is a promising strategy that warrants further evaluation in future clinical trials for malignant meningioma.

## 1. Introduction

Meningioma is the most common intracranial neoplasm in adults. Meningiomas are classified as benign (grade 1), atypical (grade 2), and anaplastic/malignant (grade 3) by the World Health Organization (WHO) classification criteria based on histological features, including cellular atypia, proliferative activity, and brain invasion [1]. Malignant meningioma accounts for approximately 2% of meningioma [2]. Even though malignant meningiomas are treated with radical surgical resection followed by radiation therapy, they frequently recur and their prognosis remains poor [3].

A number of chemotherapeutic reagents have been applied to the treatment of patients with meningioma; however, the efficacy of these reagents, including trabectedin, which was considered promising for meningioma, has mostly been small [3,4]. On the other hand, recent advances in our understanding of genomic alterations in meningioma have resulted in the introduction of potential therapeutic targets, such as mammalian target of rapamycin (mTOR), vascular endothelial growth factor receptor, the hedgehog pathway, focal adhesion kinase, AKT, and cyclin-dependent kinase [5]. Among these targets, mTOR is of particular interest because the efficacy of everolimus, an inhibitor of mTOR, for progressive meningioma was demonstrated in combination with octreotide, a somatostatin analog, in a phase II clinical trial with encouraging findings [6]. However, randomized studies are needed to confirm its efficacy in meningioma patients.

We recently reported that gemcitabine, a pyrimidine anti-metabolite chemotherapeutic drug, was very effective for high-grade meningiomas, which highly express hENT1 and dCK, a transporter and rate-limiting kinase for gemcitabine, respectively [7,8]. Accordingly, the guidelines of the European Association of Neuro-Oncology (EANO) listed gemcitabine as one of the candidate drugs for the treatment of meningioma [3]. A phase II clinical trial is currently underway after confirmation of the safety and efficacy of gemcitabine in the treatment of recurrent high-grade meningioma in a small population of patients [9]. Furthermore, we demonstrated that gemcitabine enhanced the effects of radiation together with navitoclax, an inhibitor of anti-apoptotic BCL-2 family proteins [10], suggesting a role for gemcitabine as an enhancer of treatments for malignant meningioma. Therefore, to identify drugs with anti-meningioma effects that may be enhanced by gemcitabine, we herein screened drugs that are preclinically or clinically used to treat meningioma.

## 2. Materials and Methods

### 2.1. Cell Culture

IOMM-Lee and HKBMM, human malignant meningioma cell lines, were obtained from the American Type Culture Collection (Manassas, VA, USA) and from the Riken BioResource Center (Tsukuba, Japan), respectively. Their characteristics as malignant meningioma cells were confirmed in a previous study [8]. IOMM-Lee cells were cultured in Dulbecco’s Modified Eagle’s Medium (DMEM) supplemented with 10% fetal bovine serum (FBS). HKBMM cells were cultured in Ham’s F12 medium supplemented with 10% FBS.

### 2.2. Cell Viability Assay

**Figure 1:**
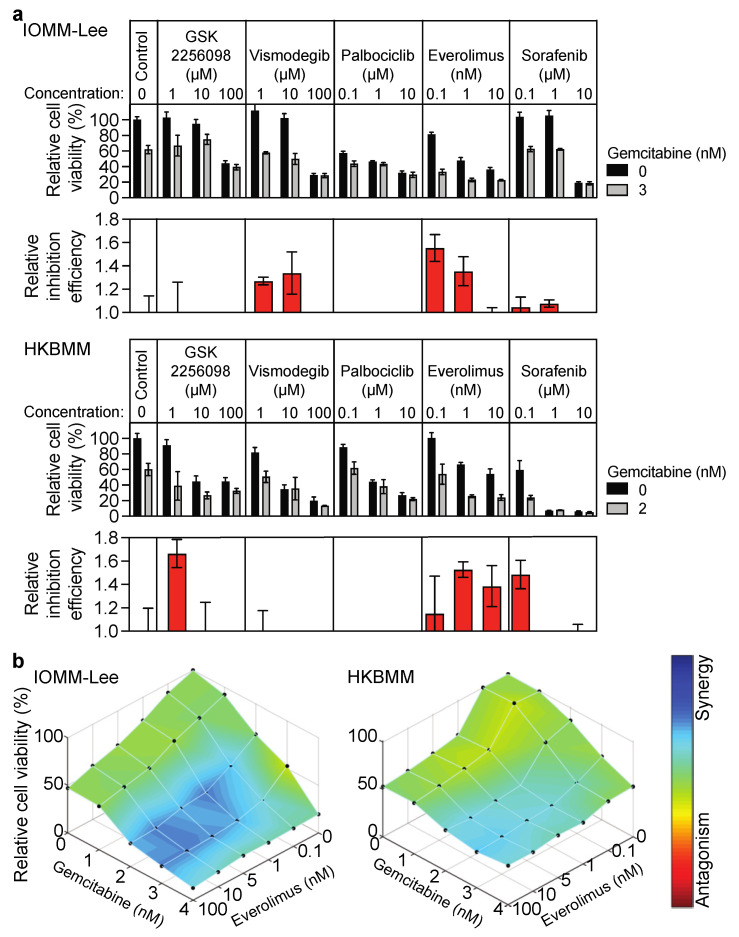
Enhancement of growth inhibitory effects of everolimus by gemcitabine in malignant meningioma cells. (**a**) IOMM-Lee and HKBMM cells plated on 96-well plates in triplicate (500 cells for IOMM-Lee and 2000 cells for HKBMM) were untreated (Control) or treated with the indicated drugs for 4 days in the absence or presence of gemcitabine (3 nM for IOMM-Lee and 2 nM for HKBMM), and cell viability was examined using the WST-8 assay. The percentages of relative cell viability to the control (gemcitabine− and drugs−) are indicated (upper), and the relative inhibition efficiency values of each drug are shown (lower). The inhibition efficiency of each drug and control (drugs−) was calculated by the following formula: 1 − [GEM+]/[GEM−], where [GEM+] and [GEM−] are the relative cell viability values of cells treated or not with gemcitabine, respectively. The relative inhibition efficiency of each drug was calculated by dividing the inhibition efficiency of each drug by that of untreated cells (drugs−). (**b**) The combination was evaluated by the WST-8 assay after 4 days in the IOMM-Lee and HKBMM meningioma cell lines. The degree of synergy was evaluated by the Loewe additivity model.

**Figure 2:**
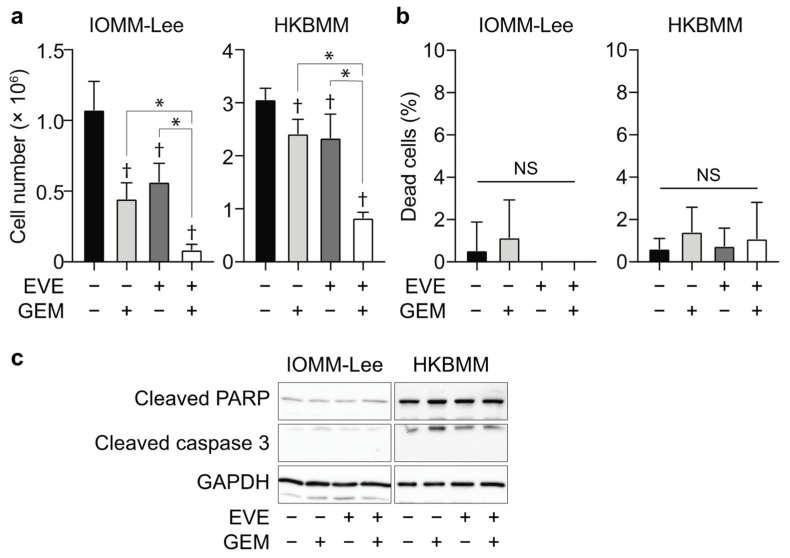
Effects of the combination of everolimus and gemcitabine on malignant meningioma cells. (**a**) IOMM-Lee and HKBMM cells plated on 6-well plates in 6 replicates (5 × 10^4^ cells per well for IOMM-Lee and 2 × 10^5^ cells per well for HKBMM) were incubated without or with everolimus (EVE, 5 nM) for 4 days in the absence or presence of gemcitabine (GEM, 3 nM for IOMM-Lee and 2 nM for HKBMM) and subjected to a cell viability assay to assess the viable cell number (**a**) and percentage of dead cells (**b**), or to an immunoblot analysis to examine the expression of the indicated proteins (**c**). Similar results were obtained from 2 independent biological replicates. Values are shown as mean + SD. *p*-values were calculated by a one-way ANOVA with Tukey’s post hoc test (**a**) or by the Kruskal–Wallis test (**b**). * *p* < 0.05. † *p* < 0.05, vs. the Control (EVE− and GEM−). NS: *p* ≥ 0.05.

Cell viability was evaluated using the WST-8 assay (Figure 1, 4a,b, and S3b,d) with Cell Counting Kit-8 (Dojindo Laboratories, Kumamoto, Japan) or the trypan blue exclusion assay (Figures 2a,b, and 4c,d) as previously described [10]. Briefly, the WST-8 reagent was added to the culture medium of cells grown on 96-well cell culture plates, and cells were incubated at 37 °C for 1–3 h. Absorbance at 450 nm was measured using a microplate reader (Model 680, Bio-Rad, Hercules, CA, USA). Relative cell viability was calculated as a percentage of the absorbance of treated samples relative to that of control samples. The degree of synergism was calculated by the Loewe additivity model, the Bliss independency model, and the highest single agent (HSA) model using Combenefit software [11]. In the trypan blue exclusion assay, after being trypsinized and suspended in phosphate-buffered saline (PBS), cells were stained with 0.2% trypan blue, and viable and dead cells were identified by their ability and inability, respectively, to exclude trypan blue. The percentage of dead cells was defined as 100 × the number of dead cells/(the number of viable cells + the number of dead cells).

### 2.3. Immunoblot Analysis

Immunoblotting was performed as previously described [12]. Cells were washed with ice-cold PBS and lysed in RIPA buffer [10 mM Tris/HCl (pH 7.4), 0.1% sodium dodecyl sulfate (SDS), 0.1% sodium deoxycholate, 1% NP-40, 150 mM NaCl, 1 mM EDTA, 1.5 mM Na_3_VO_4_, 10 mM NaF, 10 mM sodium pyrophosphate, 10 mM sodium β-glycerophosphate, and 1% protease inhibitor cocktail set III (Fujifilm Wako Pure Chemical Industries, Ltd., Osaka, Japan)]. This was followed by the immediate addition of the same volume of Laemmli buffer 2× [125 mM Tris/HCl (pH 6.8), 4% SDS, 10% glycerol] and boiling at 95 °C for 10 min. Protein concentrations were measured using a BCA protein assay kit (Thermo Fisher Scientific, Waltham, MA, USA). Samples containing equal amounts of protein were resolved by SDS-polyacrylamide gel electrophoresis and transferred to polyvinylidene fluoride membranes. Membranes were probed with a primary antibody followed by a horseradish peroxidase (HRP)-conjugated secondary antibody as recommended by the manufacturer of each antibody. Specific bands were visualized using Immobilon Western Chemiluminescent HRP Substrate (Merck Millipore, Billerica, MA, USA) and detected by the ChemiDoc Touch Imaging System (Bio-Rad).

### 2.4. Antibodies and Reagents

Anti-cleaved caspase-3 (#9661, Asp175), anti-glyceraldehyde 3-phosphate dehydrogenase (#5174, GAPDH), anti-cleaved PARP (#9541, Asp214), anti-BIM (#2819), anti-BID (#2002), anti-NOXA (#14766), anti-PUMA (#12450), anti-BAX (#5023), and anti-BAK (#12105) antibodies were purchased from Cell Signaling Technology (Beverly, MA, USA). Anti-BCL-xL (10783-1-AP) and anti-BCL-W (16026-1-AP) antibodies were purchased from ProteinTech (Rosemont, IL, USA). Anti-BCL-2 (sc-7382) and anti-MCL-1 (sc-20679) antibodies were purchased from Santa Cruz Biotechnologies (Dallas, TX, USA). Antibodies against cleaved caspase-3, BCL-2, and MCL-1 were diluted at 1:2,000. An antibody against GAPDH was diluted at 1:10,000. The other antibodies were diluted at 1:4000. Gemcitabine was purchased from Fujifilm Wako Pure Chemical Corporation and dissolved in distilled water to prepare 1 mM and 8 mg/mL stock solutions for in vitro and in vivo studies, respectively. Everolimus was purchased from LC Laboratories (Woburn, MA, USA) and dissolved in DMSO to prepare 1 mM and 100 mg/mL stock solutions for in vitro and in vivo studies, respectively. Navitoclax (ABT-263) was purchased from Chemscene (Monmouth Junction, NJ, USA) and dissolved in DMSO to prepare 100 mM and 100 mg/mL stock solutions for in vitro and in vivo studies, respectively. Bis-2-(5-phenylacetamido-1,2,4-thiadiazol-2-yl) ethyl sulfide (BPTES) was purchased from Sigma-Aldrich (St. Louis, MO, USA) and dissolved in DMSO to prepare a 10 mM stock solution. Quercetin, dasatinib, OTX015, A-1331852, A-1155463, GSK2256098, rapamycin, and temsirolimus were purchased from Cayman Chemicals (Ann Arbor, MI, USA) and dissolved in DMSO to prepare 10, 10, 1, 1, 1, 10, 100, and 100 mM stock solutions, respectively. Vismodegib, palbociclib, sorafenib, and venetoclax (ABT-199) were purchased from LC Laboratories and dissolved into DMSO to prepare 100, 5, 100, and 50 mM stock solutions. Geldanamycin was purchased from Toronto Research Chemicals (Toronto, ON, Canada) and dissolved into DMSO to prepare a 10 mM stock solution.

### 2.5. Measuring Cell Size

To quantify the size of cultured cells, phase-contrast images were obtained using a BZ-X700 microscope (Keyence, Osaka, Japan), and the area of individual cells was measured using ImageJ software [13]. Cell size was quantified in at least 40 cells per condition, and the frequency distribution of cell sizes was shown as a violin plot.

### 2.6. SA-β-Gal Staining

The SA-β-gal stain was performed using a Cellular Senescence Assay Kit (Cell Biolabs, San Diego, CA, USA) according to the manufacturer’s instructions. Briefly, cells were fixed with 0.25% glutaraldehyde for 5 min and stained with the Cell Staining Working Solution at 37 °C for 1 day. Bright-field images were obtained using a BZ-X700 microscope (Keyence, Osaka, Japan). More than 50 cells were counted to calculate the percentage of SA-β-gal-positive cells.

### 2.7. Quantitative Reverse Transcription PCR

RNA was extracted from cells using TRIzol reagent (Thermo Fisher Scientific, Waltham, MA, USA) and then reverse-transcribed into cDNA using the PrimeScript II 1st strand cDNA Synthesis Kit (Takara Bio, Kusatsu, Japan). Quantitative PCR was performed with a Thunderbird SYBR qPCR Mix (Toyobo, Osaka, Japan) using CFX96 C1000 Thermal Cycler (Bio-Rad). mRNA levels were calculated using the comparative *C*_T_ method [14] and normalized to the values of the *GAPDH* gene. cDNA was amplified by gene-specific primers (*IL1B*: forward 5′-AACAGGCTGCTCTGGGATTC-3′, reverse 5′-AGTCATCCTCATTGCCACTGT-3′; *CXCL8*: forward 5′-AAGAAACCACCGGAAGGAAC-3′, reverse 5′-ACTCCTTGGCAAAACTGCAC-3′; *CCL2*: forward 5′-CCCAGTCACCTGCTGTTATAAC-3′, reverse 5′-AGATCTCCTTGGCCACAATG-3′; *CXCL2*: forward 5′-GCAGGGAATTCACCTCAAGAAC-3′, reverse 5′-AGCTTCCTCCTTCCTTCTGG-3′; *GAPDH*: forward 5′-ACCATCTTCCAGGAGCGAGAT-3′, reverse 5′-ATGACGAACATGGGGGCATC-3′).

### 2.8. Mouse Study

After assessing cell viability using the dye exclusion method, 1 × 10^6^ viable IOMM-Lee cells suspended in 100 µL PBS were implanted into the bilateral flank regions of 5–7-week-old male BALB/cAJcl-*nu/nu* mice (CLEA Japan, Tokyo, Japan) anesthetized by a subcutaneous injection of butorphanol, midazolam, and medetomidine (5, 4, and 0.3 mg per kg body weight, respectively). Tumor volumes were assessed by measuring tumor diameters with digital calipers and calculated using the following formula: (length) × (width) × (depth) × π/6. After confirming the establishment of tumors (average tumor volumes greater than 100 mm^3^), mice were randomized according to tumor volumes and then treated with gemcitabine (dissolved in PBS, 5 or 10 mg/kg body weight, intraperitoneal injection), everolimus (dissolved in 4% DMSO in distilled water, 2 mg/kg body weight, oral gavage) navitoclax (dissolved in 20% DMSO and 80% corn oil, 100 mg/kg body weight, oral gavage), their vehicles (Control), or their combination.

### 2.9. Immunohistochemistry

Excised tissues were fixed with 4% paraformaldehyde at 4 °C overnight, embedded in paraffin, and then cut into 4 µm thick sections. After deparaffinization and rehydration, sections were treated with 3% hydrogen peroxide for 10 min. Antigens were retrieved by a heat treatment in 0.1 M Tris-HCl buffer (pH 9.0). Slides were incubated with the primary antibody at 4 °C overnight. Immunostaining was performed using Histofine Simple Stain MAX-PO (Nichirei Biosciences, Tokyo, Japan) and ImmPACT DAB (Vector Laboratories, Burlingame, CA, USA).

### 2.10. Gene Silencing by siRNA

siRNAs against human BCL-xL (*BCL2L1*: #1 HSS141361, #2 HSS141363) and Medium GC Duplex #2 of Stealth RNAi siRNA Negative Control Duplexes (non-targeting control, siControl) were purchased from Thermo Fisher Scientific. IOMM-Lee cells were transfected with siRNA against BCL-xL (siBCL-xL) or with control RNA (siControl) (50 or 2 pmol per well of a 6- or 96-well plate, respectively) using Lipofectamine RNAiMAX (Thermo Fisher Scientific, Waltham, MA, USA) according to the manufacturer’s instructions.

### 2.11. Statistics

All statistical analyses were performed using GraphPad Prism (GraphPad Software, San Diego, CA, USA). A *p* value of <0.05 was considered to be significant.

## 3. Results

### 3.1. Growth Inhibitory Effects of Everolimus on Malignant Meningioma Cells Are Enhanced by Gemcitabine

We initially tested a panel of drugs used in preclinical and clinical studies on meningioma to establish whether gemcitabine enhanced their growth inhibitory effects in the two malignant meningioma cell lines, IOMM-Lee and HKBMM. The drugs tested included GSK2256098, an inhibitor of focal adhesion kinase (NCT02523014); vismodegib, an inhibitor of the sonic hedgehog pathway (NCT02523014); palbociclib, a CDK4/6 inhibitor [15,16]; everolimus, an mTOR inhibitor [6]; and sorafenib, a multiple kinase inhibitor [17]. Among these drugs, gemcitabine consistently enhanced the effects of everolimus in both the IOMM-Lee and HKBMM malignant meningioma cell lines (Figure 1a). The Loewe additivity model, the Bliss independence model, and the HSA model indicated that this combination was synergistic in these cell lines (Figure 1b and Figure S1). Gemcitabine also enhanced the effects of other mTOR inhibitors, rapamycin and temsirolimus (Figure S2), suggesting that the enhancement by gemcitabine was mediated by the inhibition of mTOR signaling.

### 3.2. Combination of Everolimus and Gemcitabine Induces Cellular Senescence in Malignant Meningioma Cells

We then investigated the mechanisms by which gemcitabine enhanced the growth inhibitory effects of everolimus in malignant meningioma cells. In a cell viability assay, gemcitabine significantly enhanced the growth inhibitory effects of everolimus in malignant meningioma cell lines. However, it did not induce apparent cell death or activate the apoptotic pathway, indicating that the enhancement was mediated by some cytostatic mechanisms (Figure 2).

**Figure 3:**
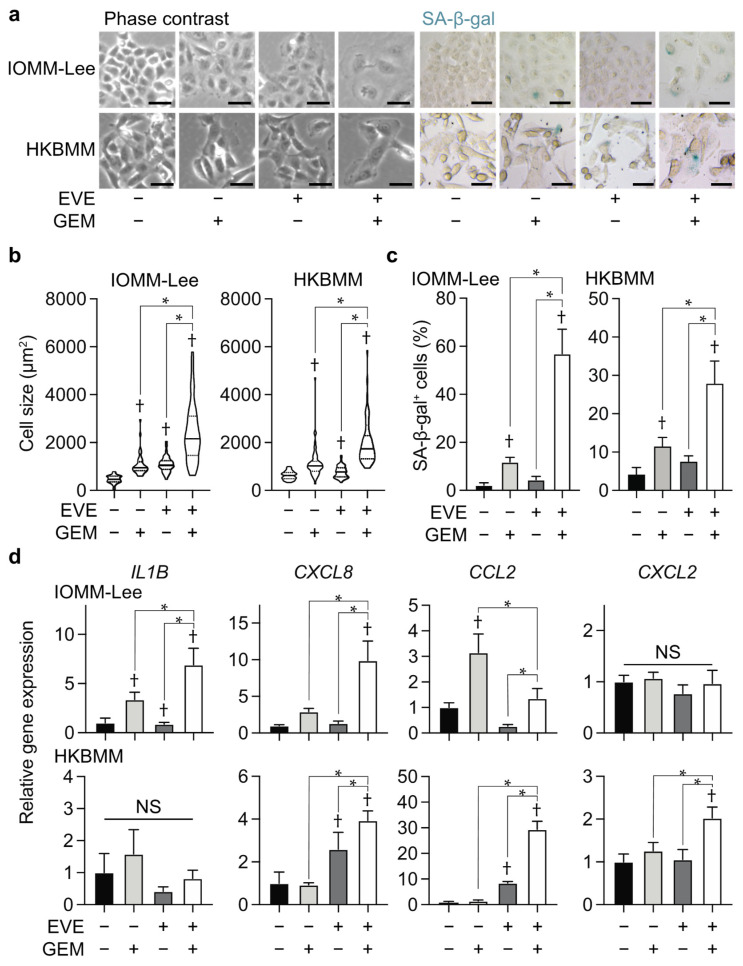
Cellular senescence in malignant meningioma cells treated with everolimus and gemcitabine. IOMM-Lee and HKBMM cells were incubated without or with everolimus (EVE, 5 nM) for 4 days in the absence or presence of gemcitabine (GEM, 3 nM for IOMM-Lee and 2 nM for HKBMM) and subjected to evaluations of cell sizes (**a**,**b**), SA-β-gal staining (**a**,**c**), and RT-qPCR in 5 replicates to assess the expression of the indicated genes (**d**). (**a**) Representative phase-contrast images and SA-β-gal staining. Scale bars, 50 µm. (**b**) Quantification of cell sizes. The size of more than 40 cells per group was measured and shown as violin plots (lines, median; dotted lines, quartile). (**c**) The percentages of SA-β-gal-positive cells were quantified (*n* = 4, each group). (**d**) Relative gene expression to untreated cells (EVE− and GEM−). Similar results were obtained from 2 independent biological replicates. Values are shown as the mean + SD. *p*-values were calculated by the Kruskal–Wallis test with Dunn’s multiple comparison test (**b**) or by a one-way ANOVA with Tukey’s (**c**) or Sidak’s multiple comparison test (**d**). * *p* < 0.05. † *p* < 0.05, vs. the Control (EVE− and GEM−). NS: *p* ≥ 0.05.

Malignant meningioma cells treated with the combination of everolimus and gemcitabine showed a flat and extended morphology with an increased cell size (Figure 3a,b). Since these characteristic morphological changes suggested that the combination of everolimus with gemcitabine induced cellular senescence, we examined senescence-associated β-galactosidase (SA-β-gal) activity, a gold standard marker for cellular senescence, in meningioma cells treated with everolimus and gemcitabine. The combination of everolimus and gemcitabine increased SA-β-gal activity in IOMM-Lee and HKBMM cells (Figure 3a,c). Furthermore, the combination of everolimus and gemcitabine increased the mRNA expression levels of inflammatory cytokines, such as *IL1B*, *CXCL8*, *CCL2*, and *CXCL2* (Figure 3d). Since increased cytokine production is one of the hallmarks of cellular senescence or senescence-associated secretory phenotypes (SASP) [18,19], these results support the induction of cellular senescence in malignant meningioma cells by the combination of everolimus and gemcitabine.

### 3.3. Navitoclax Enhances Effects of the Combination of Everolimus and Gemcitabine by Inducing Apoptosis

**Figure 4:**
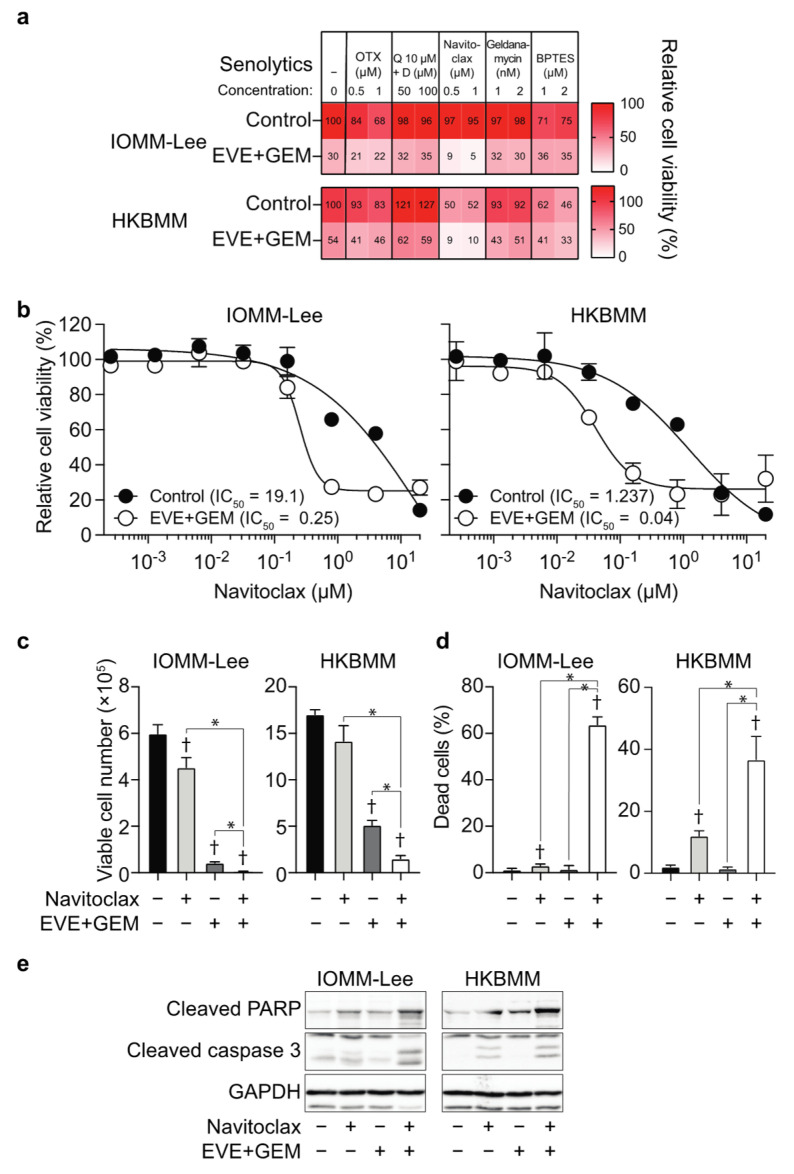
Enhancement of effects of everolimus and gemcitabine by navitoclax. IOMM-Lee and HKBMM cells plated on 96-well plates in triplicate (2000 cells per each well) were untreated (Control) or treated with everolimus (5 nM) in combination with gemcitabine (3 nM for IOMM-Lee and 2 nM for HKBMM) (EVE + GEM) for 3 days in the absence (−) or presence of indicated senolytics, and cell viability was examined using the WST-8 assay. The percentages of average relative cell viability to the Control (EVE−, GEM−, and Senolytics−) are indicated in each square and shown as heat maps (**a**). Alternatively, plated IOMM-Lee and HKBMM cells were treated with various concentrations of navitoclax for 3 days in the presence (EVE + GEM) or absence (Control) of the combination of everolimus and gemcitabine, and cell viability was examined using the WST-8 assay (**b**). IOMM-Lee and HKBMM cells plated on a 6-well plate in 6 replicates (5 × 10^4^ cells per well for IOMM-Lee and 1 × 10^5^ cells per well for HKBMM) were incubated without or with everolimus (5 nM) in combination with gemcitabine (3 nM for IOMM-Lee and 2 nM for HKBMM) (EVE + GEM) for 3 days in the absence or presence of navitoclax (1 µM) and subjected to a cell viability assay to assess the viable cell number (**c**) and percentage of dead cells (**d**). Alternatively, these cells were incubated for 2 days and subjected to an immunoblot analysis to examine the expression of the indicated proteins (**e**). Q, quercetin. D, dasatinib. Similar results were obtained from 2 independent biological replicates. Values are shown as the mean + SD. The half maximal inhibitory concentration (IC_50_) was calculated by a non-linear regression model. *p*-values were calculated by a one-way ANOVA with Tukey’s post hoc test. * *p* < 0.05. † *p* < 0.05, vs. the Control (EVE−, GEM−, and navitoclax−). Original blot images can be found at Figures S6–S8.

Previous studies reported that senolytic drugs, which selectively kill senescent cells, enhanced the effects of treatments inducing cellular senescence [20,21]. To screen for senolytic drugs that enhance the growth inhibitory effects of everolimus and gemcitabine, we treated malignant meningioma cells with senolytic drugs, such as OTX015, an inhibitor of bromodomain and extra-terminal proteins [22]; quercetin and dasatinib [23]; navitoclax, an inhibitor of anti-apoptotic BCL-2 family proteins [10,24,25]; geldanamycin, an inhibitor of heat shock protein 90 [26]; and BPTES, an inhibitor of glutaminase 1 [27], in combination with everolimus and gemcitabine. Among them, navitoclax strongly decreased the viability of malignant meningioma cells treated with everolimus and gemcitabine (Figure 4a). The treatment with everolimus and gemcitabine decreased the IC_50_ values of navitoclax by 76.4- and 30.9-fold in IOMM-Lee and HKBMM, respectively, suggesting that this combination increased the sensitivity of malignant meningioma cells to navitoclax (Figure 4b). In a cell viability assay, navitoclax significantly decreased the number of viable cells (Figure 4c) and increased the number of dead cells in everolimus- and gemcitabine-treated cells (Figure 4d) with the induction of the apoptotic markers, cleaved caspase 3, and cleaved PARP (Figure 4e). These results indicate that navitoclax enhanced the growth inhibitory effects of the combination of everolimus and gemcitabine by inducing apoptotic cell death.

### 3.4. Role for BCL-xL in Enhancements in Combined Effects of Everolimus and Gemcitabine by Navitoclax

We investigated the mechanisms by which the combination of everolimus and gemcitabine increased the sensitivity of malignant meningioma cells to navitoclax. Since navitoclax is a BH-3 mimetic that interferes with the interaction of anti-apoptotic BCL-2 family proteins with pro-apoptotic BCL-2 family proteins [28], we examined the expression of these proteins. However, the treatment with everolimus and gemcitabine did not consistently alter the expression of BCL-2 family proteins in malignant meningioma cells (Figure S3a). These results prompted us to assume that the combination of everolimus and gemcitabine increased sensitivity to navitoclax by increasing dependency on BCL-2 family proteins instead of altering protein expression. Among anti-apoptotic BCL-2 family proteins, navitoclax mainly targets BCL-2 and BCL-xL [28]. Therefore, we examined the sensitivity of malignant meningioma cells treated with gemcitabine and everolimus to the BCL-2-specific inhibitor, venetoclax, and the BCL-xL-specific inhibitors, A-1331852 and A-1155463 [28]. Everolimus and gemcitabine-treated malignant meningioma cells were sensitive to A-1331852 and A-1155463, but less sensitive to venetoclax, suggesting increased dependency on BCL-xL rather than BCL-2 (Figure S3b). To support this notion, the combination of everolimus and gemcitabine increased sensitivity to the suppression of BCL-xL expression by the siRNA treatment in malignant meningioma cells (Figure S3c,d).

### 3.5. Combined Effects of Everolimus, Gemcitabine, and Navitoclax on Malignant Meningioma Cells In Vivo

**Figure 5:**
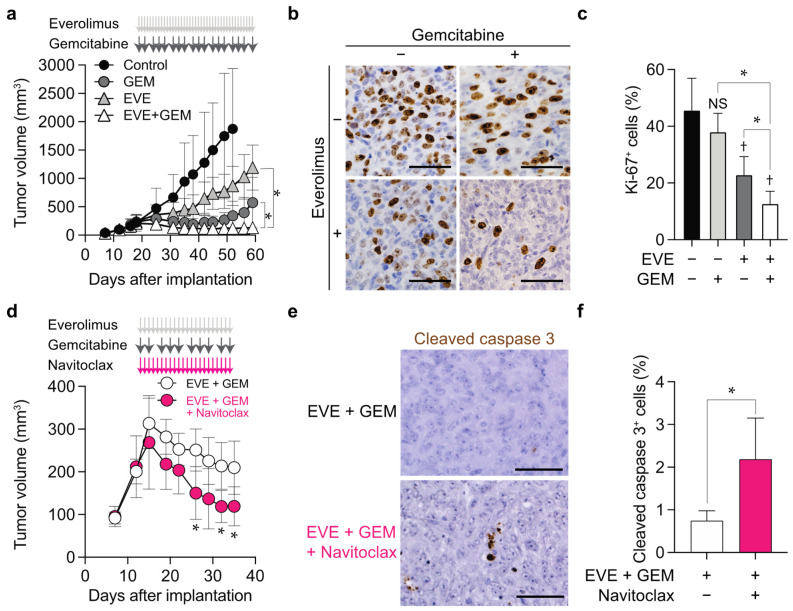
Combined effects of everolimus, gemcitabine, and navitoclax on malignant meningioma cells in vivo. IOMM-Lee cells (1 × 10^6^ cells per place) were subcutaneously implanted. After the establishment of tumors had been confirmed, mice were treated with everolimus (2 mg/kg, oral, every day) (EVE), gemcitabine (10 mg/kg, intraperitoneal injection, 3 times a week) (GEM), both (EVE + GEM), or vehicle (Control). The sizes of tumors were measured. *n* = 8, each group (**a**). Immunohistochemistry for Ki-67 in excised tumors (the Control group on day 53 and the other groups on day 60: one day after the last treatment) (**b**), and the quantification of Ki-67-positive cells (**c**). Alternatively, after the establishment of tumors had been confirmed, mice were treated with everolimus (2 mg/kg, oral, every day) in combination with gemcitabine (10 mg/kg, intraperitoneal injection, 3 times a week) and navitoclax (100 mg/kg, oral, every day) (EVE + GEM + Nav) or vehicle (EVE + GEM). The sizes of tumors were measured. *n* = 8, each group (**d**). Immunohistochemistry for cleaved caspase 3 in excised tumors (on day 36: one day after the last treatment) (**e**) and the quantification of cleaved caspase-3-positive cells (**f**). Values are shown as the mean ± or + SD. *p*-values were calculated by a one-way ANOVA with Tukey’s post hoc test (**a**,**c**), by a two-way ANOVA with Tukey’s post hoc test (**d**), or by the Student’s *t*-test (**f**). * *p* < 0.05. NS *p* ≥ 0.05 and † *p* < 0.05, vs. the Control (EVE− and GEM−). Scale bars, 50 µm.

To evaluate the potential clinical significance of the combination of everolimus, gemcitabine, and navitoclax, we examined their effects on IOMM-Lee malignant meningioma cells in a subcutaneous implantation xenograft model. The suppression of tumor growth was significantly greater with the combination of everolimus and gemcitabine than with either treatment alone in experiments with two different doses of gemcitabine (10 and 5 mg/kg body weight), and no significant weight loss was observed in mice (Figure 5a, Figures S4 and S5). An immunohistochemical analysis of Ki-67, a marker of cell proliferative activity, revealed that the combination of everolimus and gemcitabine decreased the percentage of Ki-67-positive tumor cells in mice more than either gemcitabine or everolimus alone (Figure 5b,c). Since navitoclax enhanced the effects of everolimus and gemcitabine by inducing apoptosis in vitro, we examined the therapeutic impact of adding navitoclax to everolimus and gemcitabine in vivo. The treatment with navitoclax, which alone does not exert growth inhibitory effects on meningioma xenografts [10], reduced tumor volumes by approximately 50% in mice treated with everolimus and gemcitabine (Figure 5d). Histologically, navitoclax increased the number of cleaved caspase-3-positive apoptotic cells by approximately 3-fold in vivo as well as in vitro (Figure 5e,f).

## 4. Discussion

The present study demonstrated that gemcitabine enhanced the therapeutic effects of everolimus in malignant meningioma cells both in vitro and in vivo. We also revealed that the combined therapeutic effects of these two drugs were mainly cytostatic, and that the addition of navitoclax, an inhibitor of BCL-2 family proteins, rendered these effects cytotoxic by inducing apoptosis. We recently reported the benefits of the combination of gemcitabine with radiation therapy, which has a maximum tolerated dose that limits its availability to recurrent meningiomas previously treated with radiation. The present results indicate the advantage of combination therapy with gemcitabine and everolimus because it may be used to treat patients who have already received radiation therapy.

Even with standard therapy, namely, radical surgery followed by radiation therapy, the prognosis of malignant meningioma remains poor [3]. A number of drugs, such as hydroxyurea, interferon-alpha, mifepristone, imatinib, and erlotinib, have been evaluated in clinical trials on patients with meningioma. However, the majority of clinical trials lack adequate power to prove the effectiveness of these drugs due to the small number of patients and the lack of a control arm [29]. Trabectedin, a chemotherapeutic agent used to treat advanced sarcoma, exhibited therapeutic activity for grade 2/3 meningioma cells in vitro [30]. However, a prospective randomized phase II clinical trial on patients with recurrent grade 2 or 3 meningiomas showed that trabectedin did not improve progression-free survival or overall survival [4]. A prospective phase II study on nivolumab, a programmed death 1 blocking antibody, among patients with grade 2/3 meningioma demonstrated that it failed to improve six-month progression-free survival; however, a minor subset of patients characterized by a high tumor mutation burden benefited from the treatment [31]. Clinical trials using anti-angiogenic therapy with sunitinib or bevacizumab have been performed on patients with grade 2/3 meningioma [32,33,34]. Although the findings from these uncontrolled studies are promising, they need to be confirmed in prospective controlled trials [3]. The AKT/mTOR pathway was shown to be activated in meningioma, and mTOR inhibitors suppressed meningioma cell growth in vitro and in vivo [35]. In a phase II clinical trial that evaluated the efficacy of everolimus plus bevacizumab in patients with recurrent, progressive meningioma, this combination prolonged progression-free survival slightly more in patients with WHO grade 2/3 meningioma than in patients with grade 1 meningioma [36]. Furthermore, a prospective, multicenter, single-arm, phase II study using the combination of everolimus and octreotide was performed on patients with meningioma. Although further randomized studies are warranted to confirm the findings obtained, this study revealed the anti-tumor activity and tolerability of everolimus [6]. On the other hand, a recent study on a small group of patients with meningioma documented the efficacy and safety of treatment with gemcitabine, and a phase II clinical trial is now underway [9]. As a reflection of these findings, everolimus and gemcitabine are listed as candidate drugs for meningioma in the latest version of the EANO guidelines [3]. In a phase I clinical trial on patients with solid tumors, the combination of everolimus and gemcitabine was generally tolerated well with some hematologic dose-limiting toxicities [37]. In our preclinical study, the combination of everolimus and gemcitabine was effective against malignant meningioma cells, even at a dose of gemcitabine that was lower than the clinical dose because of the high sensitivity of these cells to gemcitabine. Therefore, the present results underscore the potential impact of the combination of these two promising drugs in the treatment of malignant meningioma.

The combination of everolimus and gemcitabine synergistically enhanced their therapeutic effects in cell lines of various types of malignancies, including pancreatic cancer [38], cholangiocarcinoma [39,40], bladder cancer [41], and non-Hodgkin lymphoma [42]. The anti-tumor effects of this combination were mainly mediated by the induction of apoptosis and suppression of proliferative activity. In the present study, the combination of everolimus with gemcitabine exerted cytostatic effects rather than cytocidal effects, such as apoptosis, accompanied by the induction of cellular senescence in malignant meningioma cells. While gemcitabine reportedly induced cellular senescence in cancer cells [43], the effects of everolimus on cellular senescence are bidirectional: promoting and suppressing senescence [20]. In the present study, although everolimus alone did not result in cellular senescence, its combination with gemcitabine significantly induced cellular senescence. Similar to the present results, although rapamycin, an inhibitor of mTOR, alone did not induce senescence in the SMMC-7221 hepatocellular carcinoma cell line, its combination with 5-FU, an anti-metabolite, induced senescence and exerted synergistic anti-tumor effects [44]. These findings suggest that the induction of cellular senescence is one of the mechanisms underlying the anti-tumor effects of the combination of everolimus and gemcitabine.

The present study demonstrated that navitoclax, an inhibitor of anti-apoptotic BCL-2 family proteins, enhanced the effects of the combination of everolimus and gemcitabine in malignant meningioma cells in vitro and in vivo. The main molecular targets of navitoclax are BCL-2 and BCL-xL. In this study, the combination of everolimus and gemcitabine sensitized malignant meningioma cells to A-1331852 and A-1155463, specific inhibitors of BCL-xL, but not to venetoclax, a specific inhibitor of BCL-2, suggesting that sensitization effects were mainly dependent on the inhibition of BCL-xL. Consistent with our observations, the chemosensitization effects of navitoclax were largely dependent on the inhibition of BCL-xL in other types of malignant cells and in combination with other types of chemotherapeutics [25,45]. Cellular sensitivity to BH-3 mimetics is dictated not only by the presence of anti-apoptotic BCL-2 family proteins, but also by the activities of pro-apoptotic BCL-2 family proteins [46]. Although the dependency on BCL-xL is often caused by alterations in the expression of other BCL-2 family proteins, such as MCL-1 and NOXA [24,47,48], the combination of everolimus and gemcitabine in malignant meningioma cells did not consistently alter the expression of these proteins in the present study. Further studies are needed to elucidate the mechanisms underlying the increases in BCL-xL dependency induced by this combination in malignant meningioma cells.

In the present study, we successfully demonstrated the combined therapeutic effects of everolimus and gemcitabine in conjunction with navitoclax in subcutaneous models in vivo, which underscores the potential significance of this combination in the clinical management of malignant meningioma. We previously reported that implanted meningiomas in our intracranial model were mainly fed by brain blood vessels with a blood–brain barrier (BBB) [10], in contrast to meningiomas in patients, which are mainly fed by extra-axial blood vessels devoid of BBB. Therefore, we selected a subcutaneous model for our in vivo studies because navitoclax does not penetrate BBB [49]. To test the therapeutic effects of the combination of everolimus, gemcitabine, and navitoclax in an orthotopic setting, the development of intracranial meningioma models that more closely recapitulate the pathophysiology of meningioma is awaited.

## 5. Conclusions

The present results suggest that gemcitabine, alone or in combination with navitoclax, enhanced the anti-tumor effects of everolimus and support the clinical application of gemcitabine and navitoclax in conjunction with everolimus to the treatment of patients with malignant meningioma. Clinical trials that evaluate the impact of the combination of everolimus with gemcitabine with or without navitoclax in patients with malignant meningioma are warranted.

## Data Availability

Data are contained within the article.

## Supplementary Materials

The following supporting information can be downloaded at: https://www.mdpi.com/article/10.3390/cancers14071706/s1, Figure S1: Loewe Synergy/Antagonism score (a) of the data in Figure 1b. Analyses by the Bliss independent model (b) and HSA model (c). Figure S2: Enhancement in growth inhibitory effects of mTOR inhibitors by gemcitabine, Figure S3: Dependency of malignant meningioma cells treated with the combination of everolimus and gemcitabine on BCL-xL for their viability, Figure S4: Combined effects of everolimus and gemcitabine on malignant meningioma cells in vivo, Figure S5: Body weight of mice in the experiment in Figure 5a. Figures S6–S8: Original blot images and densitometry readings/intensity ratios of Figure 2c, Figure 4e and Figure S3a,c.
