# Supplementary material for: Gemcitabine Cooperates with Everolimus to Inhibit the Growth of and Sensitize Malignant Meningioma Cells to Apoptosis Induced by Navitoclax, an Inhibitor of Anti-Apoptotic BCL-2 Family Proteins
Source: Cancers (Basel). 2022 Mar 27;14(7):1706. doi: 10.3390/cancers14071706 (PMC8997110; doi:10.3390/cancers14071706)
Supplement: Supplementary file 1 [file cancers-14-01706-s001.zip › cancers-1602750-supplementary.pdf]

# Figure S1

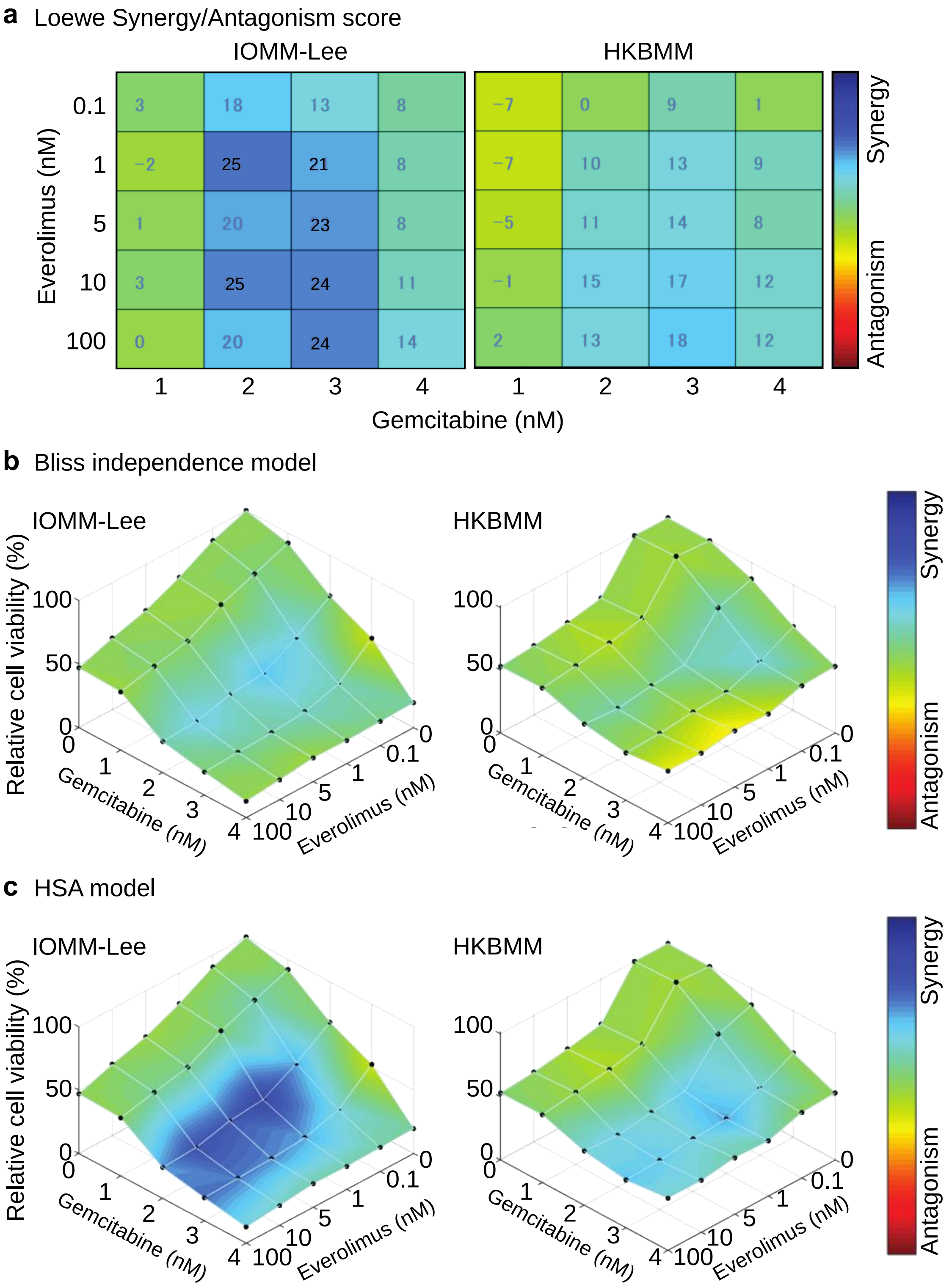


Figure S1. Loewe Synergy/Antagonism score (a) of data in Figure 1b. Analyses by the Bliss independent model (b) and HSA model (c).

## Figure S2

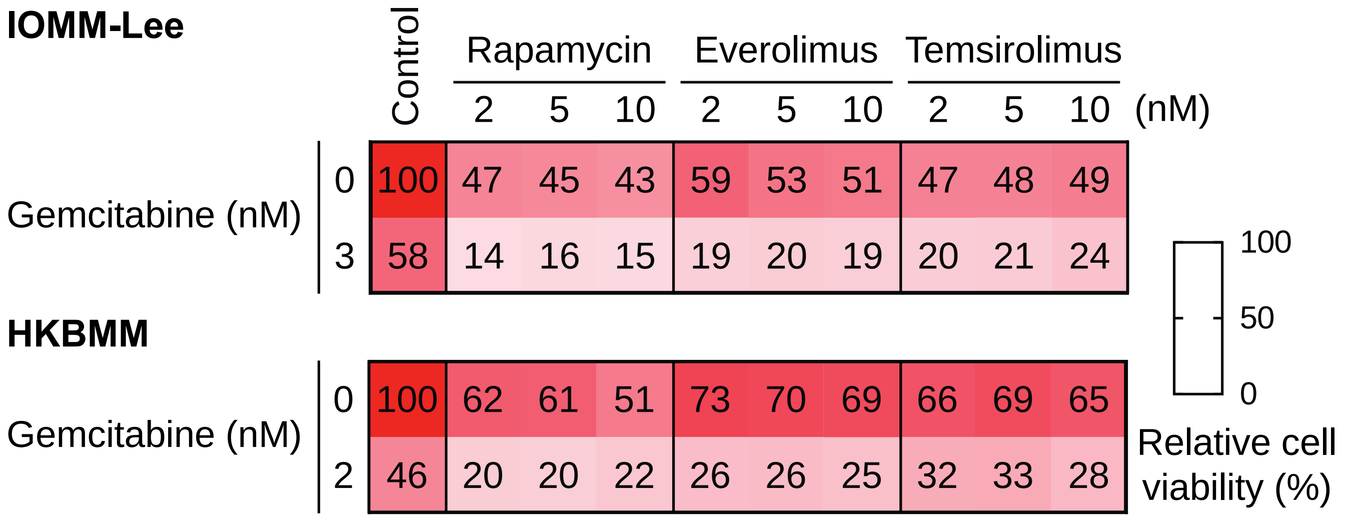


Figure S2. Enhancement of the growth-inhibitory effect of mTOR inhibitors by gemcitabine. IOMM-Lee and HKBMM cells plated on 96-well plates in triplicate (500 cells for IOMM-Lee and 2,000 cells for HKBMM) were untreated (Control) or treated with mTOR inhibitors (rapamycin, everolimus, and temsirolimus) for 4 days in the absence or presence of gemcitabine (3 nM for IOMM-Lee and 2 nM for HKBMM), and cell viability was examined using the WST-8 assay. The percentages of average relative cell viability to the control (mTOR inhibitor– and gemcitabine–) are indicated in each square and shown as heat maps.

# Figure S3

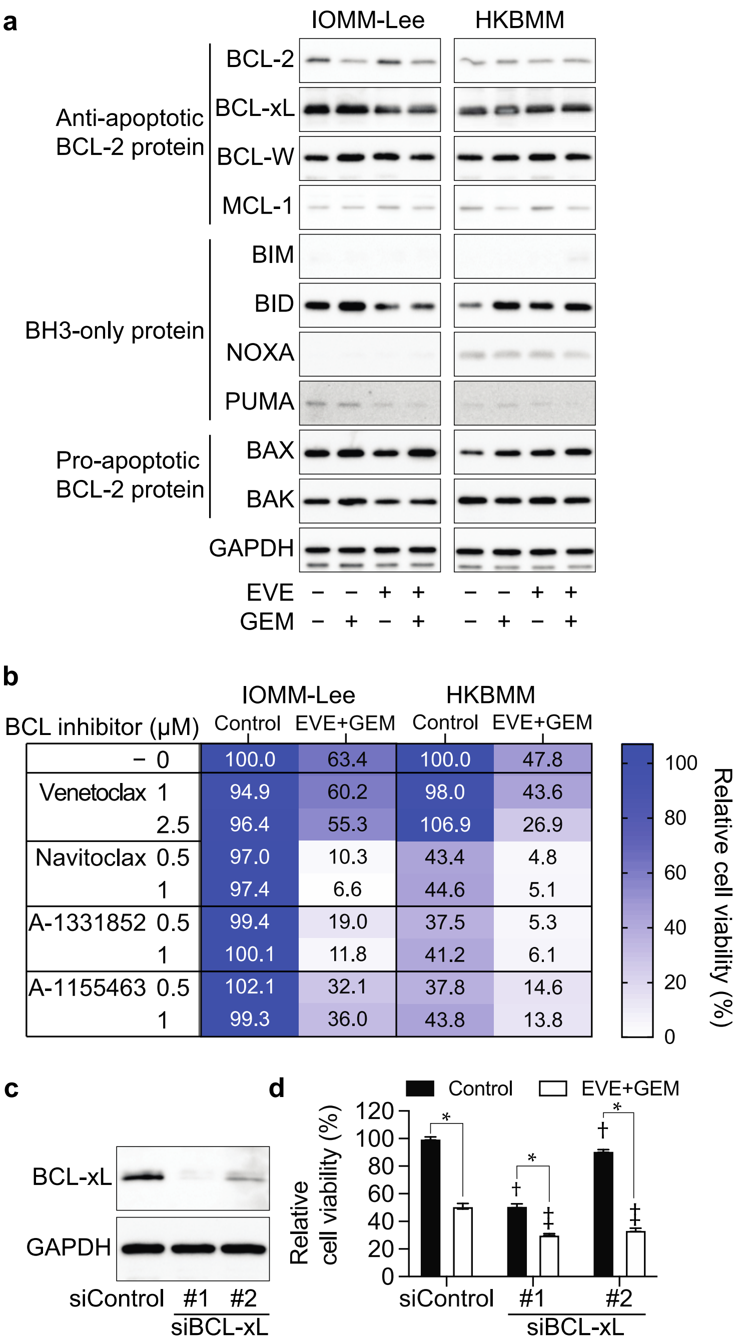


Figure S3. Dependency of malignant meningioma cells treated with the combination of everolimus and gemcitabine on BCL-xL for their viability. (a) IOMM-Lee and HKBMM cells were incubated without or with everolimus (EVE, 5 nM) for 3 days in the absence or presence of gemcitabine (GEM, 3 nM for IOMM-Lee and 2 nM for HKBMM) and subjected to an immunoblot analysis to examine the expression of the indicated proteins. (b) IOMM-Lee and HKBMM cells plated on 96-well plates in triplicate (2,000 cells per each well) were untreated (Control) or treated with everolimus (5 nM) in combination with gemcitabine (3 nM for IOMM-Lee and 2 nM for HKBMM) (EVE+GEM) for 4 days in the absence (−) or presence of inhibitors of anti-apoptotic BCL-2 family proteins (venetoclax, navitoclax, A-1331852, and A-1155463), and cell viability was examined using the WST-8 assay. (c) IOMM-Lee and HKBMM cells were transfected with control siRNA (siControl) or siRNA against BCL-xL (siBCL-xL #1 and #2) for 2 days and subjected to an immunoblot analysis to examine the expression of the indicated proteins. (d) IOMM-Lee cells plated on 96-well plates in 5 replicates were transfected with siControl or siBCL-xL (#1 and #2). On the next day, transfected cells were untreated (Control) or treated with everolimus (5 nM) in combination with gemcitabine (3 nM) (EVE+GEM) for 3 days, and cell viability was examined using the WST-8 assay. *$P < 0.05$. † and ‡, $P < 0.05$, compared with the corresponding controls (siControl-treated, EVE−, and GEM−; and siControl-treated, EVE+, and GEM+; respectively).

# Figure S4

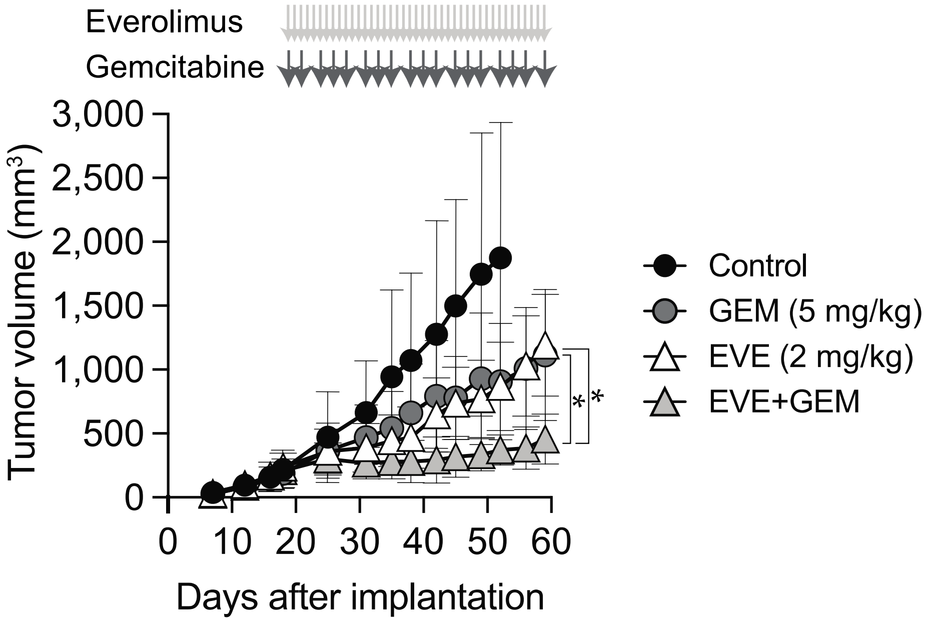


Figure S4. Combined effects of everolimus and gemcitabine on malignant meningioma cells *in vivo*. IOMM-Lee cells ($1 \times 10^6$ cells per place) were subcutaneously implanted. After the establishment of tumors had been confirmed, mice were treated with everolimus (2 mg/kg, oral, every day) (EVE), gemcitabine (5 mg/kg, intraperitoneal injection, 3 times a week) (GEM), both (EVE+GEM), or vehicle (Control). The sizes of tumors were measured. n = 8, each group. Values are shown as the mean ± SD. *P*-values were calculated by a 1-way ANOVA with Tukey's post hoc test. $^*P < 0.05$.

# Figure S5

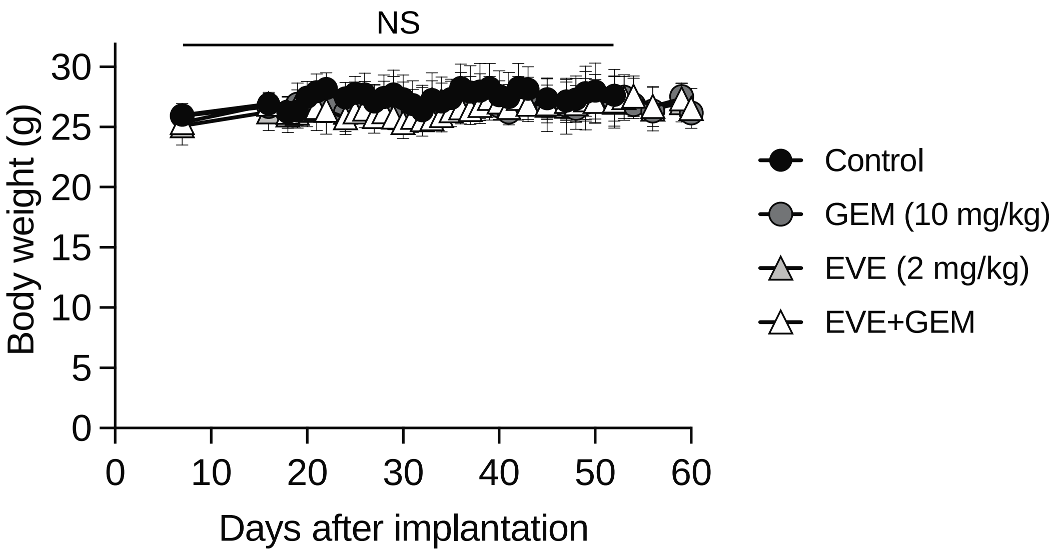


Figure S5. Body weight of mice in the experiment in Figure 5a. Values are shown as the mean ± SD. *P*-values were calculated by a 2-way ANOVA with Tukey’s post hoc test. NS, $P \geq 0.05$.

# Figure S6

## Figure 2c

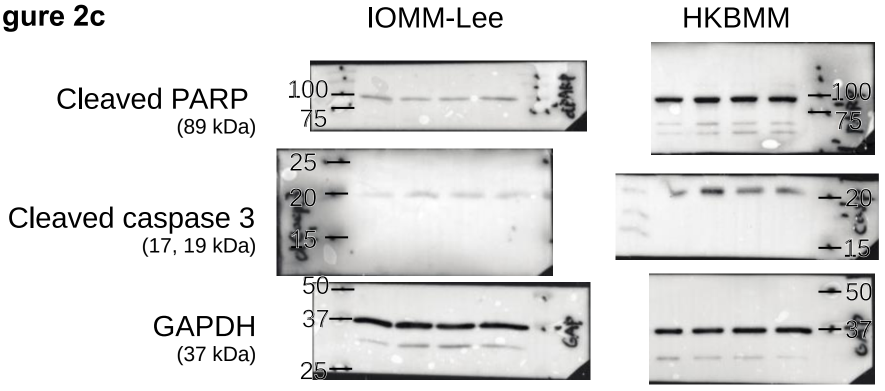


Densitometry readings/intensity ratio

| IOMM-Lee | [illegible] | [illegible] | [illegible] | [illegible] |
|---|---|---|---|---|
| GAPDH | 14763 | 15514 | 12876 | 14035 |
| Cleaved PARP | 7298 | 5775 | 5628 | 8831 |
| ratio | 0.49 | 0.37 | 0.44 | 0.63 |
| Cleaved caspase 3 | 0 | 0 | 0 | 0 |
| ratio | 0 | 0 | 0 | 0 |

| HKBMM | [illegible] | [illegible] | [illegible] | [illegible] |
|---|---|---|---|---|
| GAPDH | 9711 | 10163 | 12262 | 12934 |
| Cleaved PARP | 11101 | 13069 | 12745 | 13723 |
| ratio | 1.14 | 1.29 | 1.04 | 1.06 |
| Cleaved caspase 3 | 0 | 0 | 0 | 0 |
| ratio | 0 | 0 | 0 | 0 |

## Figure 4e

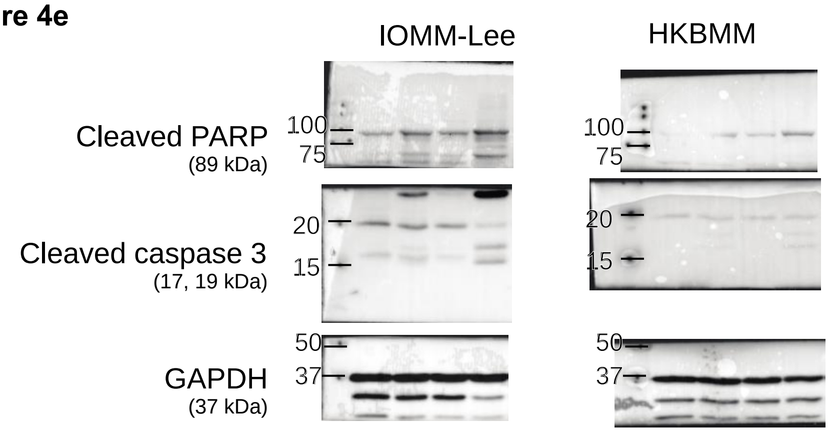


Densitometry readings/intensity ratio

| IOMM-Lee | [illegible] | [illegible] | [illegible] | [illegible] |
|---|---|---|---|---|
| GAPDH | 12238 | 12391 | 13036 | 12144 |
| Cleaved PARP | 4813 | 8259 | 6103 | 13566 |
| ratio | 0.39 | 0.67 | 0.47 | 1.12 |
| Cleaved caspase 3 | 8544 | 11828 | 4997 | 28338 |
| ratio | 0.70 | 0.95 | 0.38 | 2.33 |

| HKBMM | [illegible] | [illegible] | [illegible] | [illegible] |
|---|---|---|---|---|
| GAPDH | 15147 | 15945 | 14255 | 12337 |
| Cleaved PARP | 1279 | 3690 | 3501 | 10550 |
| ratio | 0.08 | 0.23 | 0.25 | 0.86 |
| Cleaved caspase 3 | 1398 | 9944 | 1630 | 17304 |
| ratio | 0.09 | 0.62 | 0.11 | 1.40 |

Figure S6. Original blot images and densitometry readings/intensity ratios of Figure 2c and 4e.

# Figure S7

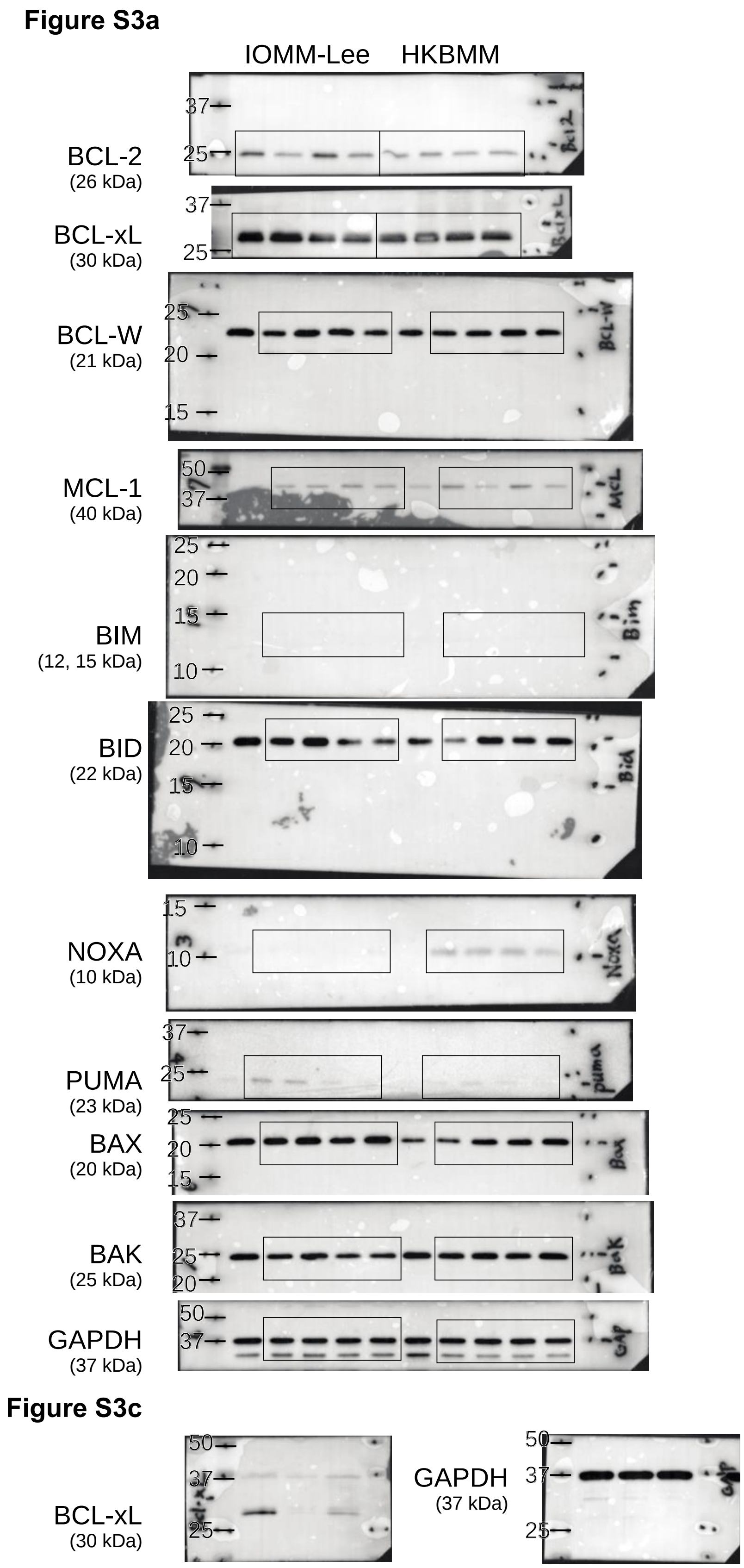


Figure S7. Original blot images of Figure S3a and S3c.

# Figure S8

## Figure S3a

Densitometry readings/intensity ratio

| IOMM-Lee | EVE−GEM− | EVE−GEM+ | EVE+GEM− | EVE+GEM+ |
|---|---|---|---|---|
| GAPDH | 12391 | 12656 | 12343 | 13129 |
| BCL-2 | 11061 | 5187 | 11749 | 6725 |
| ratio | 0.89 | 0.41 | 0.95 | 0.51 |
| BCL-xL | 18044 | 20795 | 12229 | 12192 |
| ratio | 1.46 | 1.64 | 0.99 | 0.93 |
| BCL-W | 11568 | 17162 | 15193 | 11502 |
| ratio | 0.93 | 1.36 | 1.23 | 0.88 |
| MCL-1 | 3506 | 3760 | 6081 | 4326 |
| ratio | 0.28 | 0.30 | 0.49 | 0.33 |
| BIM | 0 | 0 | 0 | 0 |
| ratio | 0.00 | 0.00 | 0.00 | 0.00 |
| BID | 19416 | 25910 | 12151 | 10758 |
| ratio | 1.57 | 2.05 | 0.98 | 0.82 |
| NOXA | 537 | 913 | 640 | 1725 |
| ratio | 0.04 | 0.07 | 0.05 | 0.13 |
| PUMA | 9114 | 7640 | 2577 | 1606 |
| raito | 0.74 | 0.60 | 0.21 | 0.12 |
| BAX | 17400 | 20769 | 15204 | 20973 |
| ratio | 1.40 | 1.64 | 1.23 | 1.60 |
| BAK | 12559 | 16156 | 10243 | 11627 |
| ratio | 1.01 | 1.28 | 0.83 | 0.89 |

Densitometry readings/intensity ratio

| HKBMM | EVE−GEM− | EVE−GEM+ | EVE+GEM− | EVE+GEM+ |
|---|---|---|---|---|
| GAPDH | 13019 | 13951 | 12819 | 11745 |
| BCL-2 | 5375 | 5565 | 4574 | 5651 |
| ratio | 0.41 | 0.40 | 0.36 | 0.48 |
| BCL-xL | 12680 | 11242 | 13645 | 14152 |
| ratio | 0.97 | 0.81 | 1.06 | 1.20 |
| BCL-W | 12118 | 13593 | 15789 | 12806 |
| ratio | 0.93 | 0.97 | 1.23 | 1.09 |
| MCL-1 | 7597 | 3285 | 8236 | 3776 |
| ratio | 0.58 | 0.24 | 0.64 | 0.32 |
| BIM | 0 | 0 | 0 | 0 |
| ratio | 0.00 | 0.00 | 0.00 | 0.00 |
| BID | 6877 | 21136 | 15974 | 19355 |
| ratio | 0.53 | 1.51 | 1.25 | 1.65 |
| NOXA | 15647 | 15044 | 14786 | 10194 |
| ratio | 1.20 | 1.08 | 1.15 | 0.87 |
| PUMA | 2550 | 2626 | 1644 | 855 |
| raito | 0.20 | 0.19 | 0.13 | 0.07 |
| BAX | 8344 | 14477 | 14877 | 16645 |
| ratio | 0.64 | 1.04 | 1.16 | 1.42 |
| BAK | 18144 | 16352 | 14294 | 17342 |
| ratio | 1.39 | 1.17 | 1.12 | 1.48 |

## Figure S3c

Densitometry readings/intensity ratio

| | siControl | siBCL-xL#1 | siBCL-xL#2 |
|---|---|---|---|
| GAPDH | 18226 | 17289 | 18362 |
| BCL-xL | 10513 | 645 | 3104 |
| ratio | 0.58 | 0.04 | 0.17 |

Figure S8. Densitometry readings/intensity ratios of Figure S3a and S3c.
